# Causal effects of inflammatory cytokines on hemangioma mediated by blood metabolites: A two-step Mendelian randomization study

**DOI:** 10.5937/jomb0-58168

**Published:** 2025-11-05

**Authors:** Yu Fan, He You Yuan, Yang Zhao Zhi, Wei Tian Zhuo, An Wang Yan, Xiao Meng, Du Zhong

**Affiliations:** 1 Department of Oral and Maxillofacial-Head and Neck Oncology, Shanghai Ninth People's Hospital, Shanghai Jiao Tong University School of Medicine, Shanghai 200011, China; 2 College of Stomatology, Shanghai Jiao Tong University, Shanghai 200011, China + National Center for Stomatology, National Clinical Research Center for Oral Diseases, Shanghai 200011, China + Shanghai Key Laboratory of Stomatology, Shanghai 200011, China

**Keywords:** hemangioma, inflammatory cytokine, metabolite, Mendelian randomization, hemangiom, inflamatorni citokin, metabolit, Mendelska randomizacija

## Abstract

**Background:**

Hemangioma is the most prevalent infantile vascular tumor. The state of inflammation and metabolism may contribute to the occurrence and development of hemangioma, but their causal relationships have not been clearly elucidated. In this study, via Mendelian randomization (MR) analysis, we aimed to investigate the causal effect of inflammatory cytokines and blood metabolites on hemangioma, and to explore the potential mediating role of metabolites.

**Methods:**

Applying large-scale genome-wide association studies (GWAS) dataset, we applied two-sample Mr to infer causal relationships among 91 inflammatory cytokines, 1091 blood metabolites and 309 metabolite ratios and hemangioma. In addition, a two-step Mr was used to assess the potential mediating role of metabolites. Functional enrichment was also performed to explore the biological pathways involved.

**Results:**

9 cytokines exhibited significant causal effects on hemangioma. Cytokines such as C-C motif chemokine 20 (CCL20), Interferon-y(IFN-y), Eotaxin and TNF-related activation-induced cytokine (TRANCE) were associated with an increased risk, while Interleukin-12 subunit beta(IL12B), C-X-C motif chemokine 11(CXCL11), Transforming growth factor-alpha (TGF-a), Oncostatin-M(OSM) and Interleukin-17A (IL17A) were inversely associated. Additionally, 52 blood metabolites and metabolite-ratios were discovered to have causal effects on hemangioma. 18 metabolites and metabolite-ratios were associated with an elevated risk of hemangioma, whereas 34 metabolites and metabolite-ratios appeared to be protective factors. Mediation analysis further identified specific metabolites, such as Gamma-glutamylvaline, as mediators in cytokine-hemangioma pathways, suggesting that they might modulate cytokine-driven tumorigenesis.

**Conclusions:**

As the first Mr study focused on hemangioma, we identified key cytokines and metabolites which might exert a causal effect on hemangioma, with several metabolites functioning as intermediators in cytokine-induced tumorigenesis process. The complex interaction between inflammation and metabolism in hemangioma was revealed, laying a foundation for future studies to explore potential targeted treatments.

## Abstract (sr)

**Uvod:**

Hemangiom je naj'češći infantilni vaskularni tumor. Stanje upale i metabolizma mogu doprineti nastanku i razvoj'u hemangioma, ali njihove uzročne veze nisu jasno razjasnjene. U ovoj studiji, putem Mendelove analize randomizacije (MR), imali smo za cilj da istražimo uzročni efekat inflamatornih citokina i metabolita u krvi na hemangiom i da istražimo potencijalnu posredničku ulogu metabolita.

**Metode:**

Primenjujući skup podataka o studijama asocijacij'e na nivou genoma (GVAS), primenili smo Mr sa dva uzorka da bismo zaključili uzročne veze između 91 inflamatornog citokina, 1091 metabolita u krvi i 309 odnosa metabolita, i hemangioma. Pored toga, Mr u dva koraka je korišćen za procenu potencijalne posredničke uloge metabolita. Takođe je izvršeno funkcionalno obogaćivanje kako bi se istražili uključeni biološki putevi.

**Rezultati:**

9 citokina je pokazalo značajne uzročne efekte na hemangiom. Citokini kao sto su hemokin 20 sa motivom C-C (CCL20), interferon-g(IFN-g), eotaksin i citokin izazvan aktivacij'om (TRANCE) povezani sa TNF-om bili su povezani sa povećanim rizikom, dok su podjedinice interleukina-12 beta(IL12B), motiv rasta C-KsmoC1(C-X-C motif chemokine 11(CXCL11), Transforming growth factor-alpha (TGF-a), Oncostatin-M(OSM) i interleukin-17A (IL17A) bili su obrnuto povezani. Pored toga, otkriveno je da 52 metabolita u krvi i omjeri metabolita imaju uzročne efekte na hemangiom. 18 metabolita i odnosa metabolita su bili povezani sa povecanim rizikom od hemangioma, dok su 34 metabolita i odnosi metabolita bili zastitni faktori. Analiza medijacije je dalje identifikovala specifične metabolite, kao sto je gama-glutamilvalin, kao medijatore u putevima citokina-hemangioma, sto sugerise da oni mogu modulirati tumorigenezu vođenu citokinom.

**Zaključak:**

Kako se prva Mr studija fokusirala na hemangiom, identifikovali smo ključne citokine i metabolite koji bi mogli imati uzročno dejstvo na hemangiom, sa nekoliko metabolita koji funkcionišu kao posrednici u procesu tumorigeneze izazvane citokinom. Otkrivena je kompleksna interakcija između upale i metabolizma kod hemangioma, postavljajuci osnovu za buduce studije za istraživanje potencijalnih ciljanih tretmana.

## Introduction

Hemangioma is the most prevalent infantile tumor, with a reported incidence range from 5% to 10%. Hemangioma typically presents as a red or purple elevated lesion after birth, most frequently on the facial and cervical skin, with variations in size and appearance [1]. Although this is a benign vascular tumor, it might develop several serious complications, such as ulceration, bleeding, permanent facial disfigurement, or even life-threatening situation. Currently, propranolol is the first-choice treatment for hemangioma, while clinicians may sometimes recommend surgery or laser therapy [2]. However, for complex or extremely serious hemangioma, there is no current treatments based on etiology, which highlights the critical need for a deep understanding of pathogenic mechanism.

Excessive angiogenesis is the most prominent histological feature in hemangioma lesions. And abnormal inflammatory microenvironment is closely related to angiogenesis and tumorigenesis. Influenced by prolonged inflammatory signaling, hypoxia, and altered metabolite levels, chronic inflammation leads to tissue injury, epithelial mutagenesis, endothelial dysfunction and angiogenesis, ultimately resulting in tumor initiation or progression [3]. As a vascular tumor, countless inflammatory cytokines secreted into the blood could directly influence lining endothelial cells (ECs). However, the causal role of inflammatory factors in hemangioma remains unclear. Besides, vascular ECs metabolism was recently recognized as a driving force of angiogenesis, and targeting ECs metabolism has emerged as a promising strategy for normalizing ECs dysfunction [4]. Therefore, it is significant to find the causal connection among inflammatory cytokines, metabolites, and hemangioma.

Current studies on hemangioma pathogenesis rely predominantly on observational study or in vitro models, which are confounded by reverse causation and limited in pediatric populations. Mendelian randomization (MR) leveraging genetic variants as instrumental variables, offers a robust framework to infer causal relationship. MR is a methodology that employs genetic variations associated with exposures to assess potential causal relationships with outcomes. MR rests on 3 main assumptions [5]: 1) the genetic variant is related to the exposures; 2) the genetic variant is not connected with confounders; and 3) the genetic variant influences the outcome only through the exposure. The genetic variants typically are single nucleotide polymorphisms (SNPs). However, no prior MR study has systematically investigated the roles of inflammatory cytokines and metabolites in hemangioma, leaving their causal interplay unexplored.

In this study, via two-sample MR analysis, we aimed to investigate the causal role of inflammatory cytokines and blood metabolites in hemangioma. Furthermore, we employed two-step mediation MR analysis to investigate intermediary casual effect of metabolites. As the first Mendelian randomization study concentrating on hemangioma, our findings may provide novel insights into the molecular regulatory relationships of hemangioma, laying a foundation for future studies to explore targeted treatments.

## Materials and methods

### Research design

**Figure 1:**
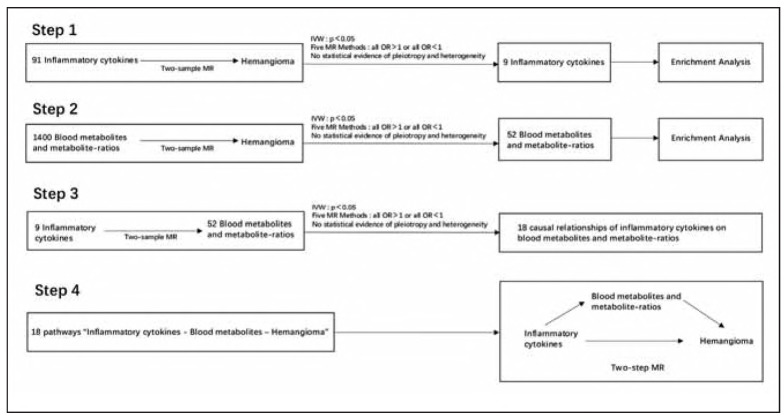
Flow chart of the analysis. In step 1, a two-sample MR analysis was conducted to assess the causal relationship between inflammatory cytokines and hemangioma. In step 2, we identified the blood metabolites and metabolite-ratios with causal effect on hemangioma. In step 3, we further explored potential causal relationships of inflammatory cytokines on blood metabolites and metabolite-ratios identified before. In the last step 4, for each identified cytokine-metabolite-hemangioma pathway, a two-step MR was applied to assess the mediating effects of metabolites. Five MR Methods: Inverse variance weighted (IVW), Weighted median, MR-Egger, Simple mode, Weighted mode.

This study investigated the causal relationship between blood inflammatory cytokines and hemangioma, with metabolites serving as potential mediators. Our analysis involved four primary steps (Figure 1). In step 1, a two-sample MR analysis was conducted to assess the causal relationship between inflammatory cytokines and hemangioma. In step 2, we identified the blood metabolites and metabolite-ratios with causal effect on hemangioma. In step 3, we further explored potential causal relationships of inflammatory cytokines on blood metabolites and metabolite-ratios identified before. Following this, in the last step, for each identified cytokine-metabolite-hemangioma pathway, a two-step MR was applied to assess the mediating effects of metabolites.

### Data source

The genetic dataset of 91 inflammatory cytokines was obtained from a recent study [6], which included 14,824 participants of European participants. And the genetic dataset of 1091 blood metabolites and 309 metabolite ratios was sourced from a large-scale GWAS study [7] involving a cohort of 8299 individuals of European ancestry. The hemangioma GWAS data was obtained from the FinnGen database [8], consisting of genetic information from 2846 cases and 450887 controls in European. All the GWAS summary data for each cytokine and metabolites are publicly accessible from GWAS catalog.

### Selection of instrumental variables

The genetic instrumental variables (IVs) used in this study met three core assumptions [5]: 1) the genetic variant is related to the exposures; 2) the genetic variant is not connected with confounders; and 3) the genetic variant influences the outcome only through the exposure. We applied a linkage disequilibrium threshold of *r^2^* < 0.001 within a 1000 kb window, along with a clustering criterion of *p* < 1 X 10^-5^. To maintain consistency, we harmonized SNP effect sizes on both the exposure and outcome by aligning beta values to the same alleles. As well, all palindrome SNPs and repeated SNPs were excluded. *F* statistics were calculated to assess instrument strength, with *F* > 10 indicating robust instruments. Instruments with *F* < 10 were excluded to prevent weak instrument bias.

### MR statistical analyses

All Mendelian randomization analyses of this research were performed by R software (Version 4.4.1). We used two-sample MR to demonstrate whether exposure had a causal effect on outcome. The Inverse variance weighted (IVW) method was applied as the primary method. Meanwhile, we also applied other four methods to assess the causal relationships: Weighted median, MR-Egger, Simple mode, and Weighted mode. In our study, an exposure was considered to have a statistically significant causal effect on the outcome if it met the following criteria: the *p*-value calculated by IVW is less than 0.05; the odds ratio (OR) derived from all five methods mentioned before are either all greater than 1 or all less than 1; there is no statistical evidence of pleiotropy and heterogeneity.

### Mediation analysis

We performed a two-step Mendelian randomization mediation analysis to assess the causal effect of inflammatory cytokines (exposure) on hemangioma (outcome) mediated through metabolites (mediators). Genetic variants were employed as IVs to estimate the causal effect of inflammatory cytokines on metabolites (beta1), and subsequently, the effect of metabolites on hemangioma (beta2). Also, we evaluated the total effect of inflammatory cytokines on hemangioma. The total effect was decomposed into direct effect and mediated effect, the latter being mediated through metabolites (Mediated effect = beta1 * beta2). The mediated proportion was calculated as the ratio of the mediated effect to total effect. At the end, the standard error and 95% confidence interval of the mediated effect were calculated, and statistical significance was evaluated using Z-test.

### Heterogeneity, horizontal pleiotropy and sensitivity analysis

We conducted rigorous assessments of heterogeneity and horizontal pleiotropy to ensure the robustness and reliability of our MR analyses. Heterogeneity was evaluated using Cochran's Q test, where a significant *p*-value suggests inconsistent SNP effects, potentially indicating varying pathways through which SNPs influence the outcome. Horizontal pleiotropy was assessed by the MR-Egger intercept test. The MR-Egger intercept test examines the intercept for significant deviations from zero, suggesting pleiotropic SNPs that may affect the outcome independently of the exposure, thus introducing bias. To confirm the stability of results, we performed some sensitivity analyses. Single SNP analysis was used to evaluate the influence of individual SNPs, identifying variants with disproportionate impacts. Leave-one-out analysis assessed the robustness of the results by sequentially excluding each SNP, allowing us to check for result consistency. We also applied five methods (IVW, Weighted median, MR-Egger, Simple mode, Weighted mode) to assess the potential causal rela tionships and made corresponding scatter plots. In brief, combination of heterogeneity, pleiotropy, and sensitivity analyses provided a comprehensive valida tion framework, enhancing the reliability of our causal inferences.

### Enrichment analysis

Kyoto Encyclopedia of Genes and Genomes (KEGG) enrichment analysis on the inflammatory cytokines causally related to hemangioma was performed by R package ClusterProfiler. Blood metabolites pathway enrichment analysis was performed by MetaboAnalyst 6.0 website platform [9] utilizing the RaMP-DB set library, which integrates 3,694 metabolite and lipid pathways from KEGG via HMDB, Reactome, and WikiPathways.

## Results

### 9 inflammatory cytokines were identified to have causal effects on hemangioma

**Figure 2:**
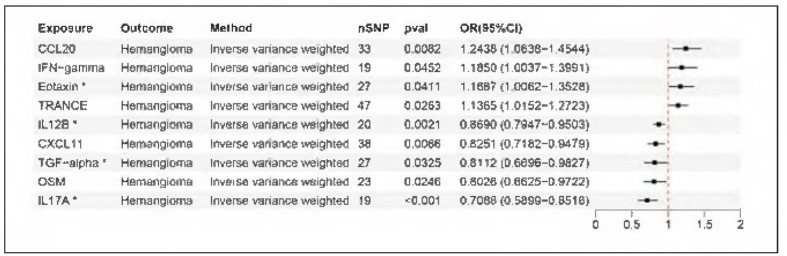
Forest plot of causal associations of inflammatory cytokines with hemangioma. This figure displayed is the result analyzed via the classic IVW method. Asterisk mark means p-value<0.05 identified by at least three of five MR analysis methods (IVW, Weighted median, MR-Egger, Simple mode, Weighted mode). OR > 1 means exposure increases the risk of developing the outcome; OR < 1 means exposure decreases the risk of developing the outcome.

Among 91 inflammatory cytokines, 9 cytokines were identified to have a significant causal effect on hemangioma. As shown in Figure 2, forest plots demonstrated the causal effect and confidence intervals derived from the IVW method. For each cytokine, the OR derived from all five methods (IVW, Weighted Median, MR-Egger, Simple Mode, and Weighted Mode) were consistently either all greater than 1 or all less than 1. Among 9 identified cytokines, 4 cytokines were positively associated with hemangioma: C-C motif chemokine 20 (CCL20, OR= 1.2438, *p* = 0.0062), Interferon gamma (IFN-γ, OR=1.1850, *p*=0.0452), Eotaxin (OR=1.1667, *p* = 0.0411) and TNF-related activation-induced cytokine (TRANCE, OR=1.1365, *p* = 0.0263). Besides, there were 5 cytokines negatively associated with hemangioma: Interleukin-12 subunit beta (IL12B, OR=0.8690, *p*=0.0021), C-X-C motif chemokine 11 (CXCL11, OR=0.8251, *p*=0.0066), Transforming growth factor-alpha (TGF-α, OR=0.8112, *p*=0.0325), Oncostatin-M (OSM, OR=0.8026, *p*=0.0246), Interleukin-17A (IL17A, OR=0.7088, *p*<0.001) (Figure 2). Notably, Eotaxin, IL12B, TGF-α and IL17A showed higher evidence related to hemangioma, having *p*-values< 0.05 in at least three of five MR methods. Cochran's Q test for all 9 cytokines revealed no evidence of heterogeneity, and the MR-Egger intercept test indicated no horizontal pleiotropy. Single SNP analysis and Leave-one-out analysis demonstrated the robustness and reliability.

### 52 blood metabolites and metabolite-ratios were identified to exert causal effects on hemangioma

**Figure 3:**
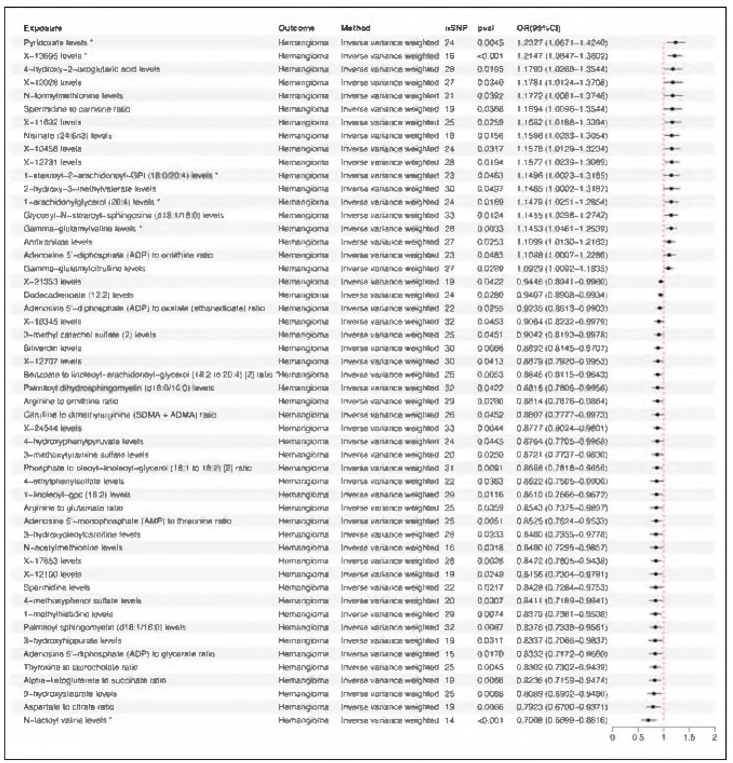
Forest plot of causal associations of blood metabolites and metabolite-ratios with hemangioma. This figure displayed is the result analyzed via the classic IVW method. Asterisk mark means p-value<0.05 identified by at least three of five MR analysis methods (IVW, Weighted median, MR-Egger, Simple mode, Weighted mode). OR > 1 means exposure increases the risk of devel oping the outcome; OR < 1 means exposure decreases the risk of developing the outcome.

According to screening criteria, 52 out of 1400 blood metabolites and metabolite-ratios were identified to have significant causal effects on hemangioma (Figure 3). MR analysis results demonstrated that 18 blood metabolites and metabolite-ratios were associated with an increased risk to hemangioma (OR>1). Pyridoxate showed the highest OR (OR=1.2327, *p*=0.0045). Meanwhile, 34 blood metabolites and metabolite-ratios were found to be associated with a decreased risk to hemangioma (OR<1), among which N-lactoyl valine had the smallest OR (OR=0.7008, *p*<0.001). For each metabolite and metabolite-ratio identified, the OR derived from all five methods (IVW, Weighted Median, MR-Egger, Simple Mode, and Weighted Mode) were consistently either all greater than 1 or all less than 1. Sensitivity analyses confirmed that all identified metabolites and ratios were free of horizontal pleiotropy and heterogeneity. Single SNP analysis and Leave-one-out analysis demonstrated the robustness and reliability.

### Functional enrichment analysis of hemangioma-related inflammatory cytokines and blood metabolites

**Figure 4:**
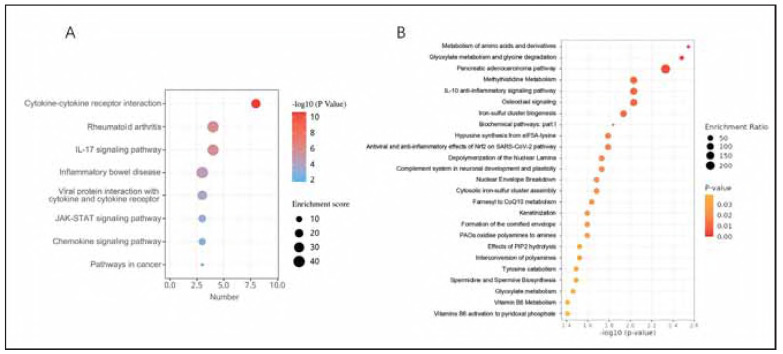
Diagram of functional enrichment analysis of inflammatory cytokines and blood metabolites. (A) KEGG enrichment analysis for hemangioma-related inflammatory cytokines. (B) Enrichment analysis of blood metabolites related to hemangioma.

To further understand the broader biological pathways involved, we conducted a functional enrichment analysis of inflammatory cytokines and blood metabolites. According to the KEGG enrichment results for hemangioma-related inflammatory cytokines (Figure 4A), highlighting pathways such as cytokine-cytokine receptor interaction, rheumatoid arthritis, and IL-17 signaling pathway, have potential implications in inflammatory and hemangioma occurrence. On the other hand, as for blood metabolites associated with hemangioma, enriched pathways were mainly metabolism of amino acids and derivatives, glyoxylate metabolism and glycine degradation and so on (Figure 4B).

### Alteration of several blood metabolites or metabolite ratios could modulate the effects of inflammatory cytokines in hemangioma

Via MR analysis, 18 causal relationships between inflammatory cytokines and blood metabolites or metabolite ratios were identified. Also, we did rigorous sensitivity analysis, single SNP analysis and Leave-one-out analysis to demonstrate the robustness and reliability. To further investigate the potential mediating role of blood metabolites in the causal pathway from inflammatory cytokines to hemangioma, we conducted a two-step Mendelian randomization mediation analysis. We found that some cytokines (CXCL11, IFN-γ, IL12B, IL17A, OSM) were mediated by more than one metabolite. However, for Eotaxin and TGF-α, only one respective metabolite-mediated pathways was identified. Among all mediating pathways, the highest level of evidence was for pathway TGF-α - Gamma-glutamylvaline - hemangioma, as the *p*-value of Z-test is less than 0.05. Specifically, the total effect of TGF-α on hemangioma is -0.2093, while the mediated effect of Gamma-glutamylvaline is 0.0218 (95%CI 0.00216, 0.0414) with a mediated proportion of -10.4% (95%CI -1.03%, - 19.8%). Our results indicate that, alteration of several blood metabolites or metabolite ratios, could modulate the effects of inflammatory cytokines on hemangioma, either resisting or promoting hemangioma development.

## Discussion

Hemangioma, as a kind of vascular tumor, can be directly affected by abundant inflammatory factors and metabolites in blood. Inflammation is often perceived as an incubator of the tumor microenvironment [3]. Via MR analysis, we identified four cytokines that might have a positive causal effect on hemangioma: Eotaxin, CCL20, IFN-γ and TRANCE (Figure 2). Eotaxin induces angiogenic responses by CCR3+ ECs [10]. Via binding to the CCR6 receptor, CCL20 could target ECs directly and enhance vessel formation [11]. As for TRANCE, it could stimulate DNA synthesis, chemotactic motility of ECs and promote new vessel formation [12]. Nevertheless, the role of IFN-γ in hemangioma is still controversial. In a pilot study [13], high level IFN-γ was detected in serum from patients carrying multiple hemangioma lesions. Conversely, another study [14] showed that IFN-γ effectively inhibited hemangioma proliferation in a mouse model.

Besides cytokines as risk factors, we also identified five cytokines (IL12B, TGF-α, IL17A, CXCL11, OSM) that exhibited negative associations with hemangioma (Figure 2). IL12B is a key component of interleukin-12, which exerts antitumor effects on both solid tumors and hematological malignancies by inhibiting angiogenesis [15]. CXCL11, through interaction with the chemokine receptor CXCR3, ability of tube formation is decreased and vascular patterning is negatively affected [16]. TGF-α could induce endothelial-to-mesenchymal transition and promote hemangioma regression [17]. As for OSM and IL17A, modulatory effects for vascular biological process are still controversial. OSM could exhibit a protective effect in acute ischemic injury models [18]. Conversely, OSM also has a deteriorating effect on endothelial dysfunction associated with atherosclerosis [19]. IL17A, a pro-inflammatory cytokine produced by Th17 cells, has complicated regulatory effects on vascular network. On one hand, IL17A can enhance VEGF-dependent angiogenesis in retinal neovascularization lesions [20]. On the other hand, IL-17A can promote aging of ECs by activating the JNK signaling pathway and upregulating expression of FTO [21].

During proliferating phase of hemangioma, sprouting needs huge supply of energy, which requires glycolysis, amino acid metabolism and fatty acid oxidation. Glycolysis is crucial in the pathogenesis of hemangioma, arising in response to localized tissue hypoxia [1]. Metabolite functional enrichment analysis identified that hemangioma-associated metabolites were enriched in some pathways linked to glycolysis , such as glyoxylate metabolism and glycine degradation (Figure 4B). Besides glucose metabolism, lipid metabolism is also crucial in hemangioma. We found plenty of lipid metabolites related to hemangioma. Among these molecules, 1-stearoyl-2-arachidonoyl-GPI (18:0/20:4) and 1-arachidonyl-glycerol (20:4) had high MR evidence, which are risk factors for hemangioma (Figure 3). Protein and amino acid metabolism also participate in hemangioma pathogenesis. Among all the metabolites related to hemangioma, N-lactoyl valine, an amino acid metabolite, was found to have the smallest OR (OR=0.7008, *p* 0.001) (Figure 3), indicating it might be a protective factor.

**Figure 5:**
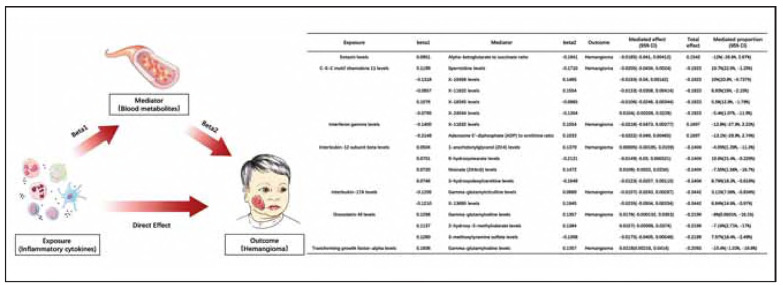
Potential roles of inflammatory cytokines and blood metabolites associated with hemangioma. Both blood metabolites and inflammatory cytokines influence hemangioma, whilst blood metabolites act as mediating factors in the process. beta1: the effect of inflammatory cytokines on blood metabolites and metabolite-ratios; beta2: the effect of blood metabolites and metabolite-ratios on hemangioma; Mediated effect: the effect of inflammatory cytokines on hemangioma through affecting blood metabolites; Mediated effect = beta1 * beta2; Total effect: the total effect of inflammatory cytokines on hemangioma; Mediated proportion = (Mediated effect / Total effect)*100%; beta>0 means positive causal effect, beta<0 means negative causal effect.

Although numerous metabolites are associated with hemangioma, we propose that metabolites may play more of an intermediary role in hemangioma, because most metabolites are positioned midstream or downstream in biological processes. Through two-step MR mediation analysis, we identified key cytokine-metabolite-hemangioma pathways, as metabolites may either enhance or mitigate the potential effects of cytokines (Figure 5). For example, the TGF-α to Gamma-glutamylvaline exhibited a significant mediated effect with a mediated proportion of -10.4%, suggests that Gamma-glutamylvaline may counteract TGF-α's impact on hemangioma.

## Conclusion

In conclusion, this is the first study to apply Mendelian randomization method to investigate hemangioma. We combined with a large sample size MR analyses and rigorous sensitivity analyses, providing insights into the causal roles of inflammatory cytokines and metabolites in hemangioma. Furthermore, our mediation analysis highlights potential intermediary roles of metabolites. The complex interaction between inflammation and metabolism in hemangioma preliminarily revealed in this study, is a foundation to explore potential targeted treatments. We might inhibit or enhance some target cytokines for disrupting pathological angiogenesis of hemangioma. And Gamma-glutamylvaline supplementation offers a prophylactic strategy for high-risk newborns. The protective metabolite N-lactoyl valine could serve as a plasma biomarker for early intervention, and integrating metabolite modulators with propranolol may enhance efficacy.

However, this study still has some limitations. The GWAS study population is European ancestry, limiting the generalizability. Additionally, the availability of genetic instruments for some cytokines and metabolites were limited, potentially impacting the power to detect more subtle causal effects. Finally, there is a shortage of large-scale clinical trials or basic research.

## Dodatak

### Funding

This work was supported and funded by National Natural Science Foundation of China (Grant No. 82201085 and No. 81870780) and Natural Science Foundation of Shanghai (Grant No. 23ZR1437900).

### Acknowledgements

We want to acknowledge the participants and investigators of the FinnGen study. And we greatly appreciate that Jing Hua Zhao and Yi Heng Chen shared the GWAS data included in the study. Besides, we are grateful to Zhi Qiang Pang developing MetaboAnalyst 6.0 platform.

### Data Availability

The data that support the findings of this study are available from the corresponding author upon reasonable request.

### Author Contributions

Study Design: FY, YHY, ZD; Data Curation: FY, YHY, ZYZ; Formal Analysis: FY, ZWT; Funding Acquisition: ZD, YAW; Methodology: Mx, YHY: Software: FY, YHY: Supervision: ZD, YAW; Visualization: FY, ZD; Writing - Original Draft Preparation: FY ZD; Writing - Review and Editing: FY, YAW, ZD. All authors read and approved the final manuscript.

### Ethic Statement

Not applicable.

### Conflict of interest statement

All the authors declare that they have no conflict of interest in this work.

Fan Yu, Yuan He You, Zhi Yang Zhao, These authors have contributed equally and should be con sidered as co-first authors.
